# Depression, Anxiety and Sleep Alterations in Caregivers of Persons With Dementia After 1-Year of COVID-19 Pandemic

**DOI:** 10.3389/fpsyt.2022.826371

**Published:** 2022-02-10

**Authors:** Cinzia Bussè, Teresa Barnini, Milena Zucca, Innocenzo Rainero, Stefano Mozzetta, Andrea Zangrossi, Annachiara Cagnin

**Affiliations:** ^1^Department of Neuroscience (DNS), University of Padova, Padova, Italy; ^2^Department of Neuroscience “Rita Levi Montalcini, ” Aging Brain and Memory Clinic, University of Torino, Torino, Italy; ^3^Padova Neuroscience Center, University of Padova, Padova, Italy

**Keywords:** dementia, COVID, lockdown, burden, caregivers, depression

## Abstract

**Background:**

Social isolation due to COVID-19 pandemic has an important psychological impact particularly in persons with dementia and their informal caregivers.

**Aim:**

To assess frequency and severity of long-term stress-related symptoms in caregivers of patients with dementia 1-year after the beginning of COVID-19 pandemic and to identify predictors of psychological outcomes.

**Methods:**

Eighty-five caregivers were involved in a longitudinal study with 1-year follow-up during pandemic in Italy. At baseline in April 2020 a telephone interview assessed socio-demographic characteristics of caregivers and self-perception of distress symptoms. After 1 year, between March and April 2021, the same standardized interview was delivered to the caregivers' sample. In addition, scales assessing levels of depression and anxiety (DASS-21), sleep disturbances (PSQI) and coping strategies (COPE-NVI) were administered to the caregivers and to 50 age and sex-matched non-caregivers subjects. Linear regression analysis was performed to investigate the power of baseline variables to predict long-term psychological outcomes.

**Results:**

After 1 year of pandemic frequency of caregivers' stress-related symptoms increased respect to baseline: depression (60 vs. 5, 9%; *p* < 0.001), anxiety (45, 9 vs. 29, 4%; *p* = 0.035), irritability (49, 4 vs. 24, 7%; *p* < 0.001), and anguish (31, 7 vs. 10, 6%; *p* < 0.001). Frequency of severe depression was higher in caregivers than in non-caregivers (*p* = 0.002) although mean levels of depression were comparable in the two groups. Long-term higher depression was predicted by a model built on baseline information (r^2^ = 0.53, *p* < 0.001) where being female (t = −3.61, *p* < 0.001), having lower education (t = −2.15, *p* = 0.04), presence of feelings of overwhelm (t = 2.29, *p* = 0.02) and isolation (t = 2.12, *p* = 0.04) were significant predictors. Female sex was also predictive of anxiety (t = −2.7, *p* = 0.01) and poor sleep quality (t = −2.17, *p* = 0.03).

**Discussion:**

At 1 year follow-up caregivers of patients with dementia reported higher prevalence of all stress-related symptoms respect to the acute phase of lockdown, particularly depression. Long-lasting stressful conditions may cause exhaustion of resilience factors and increased depression. Planning interventions should support caregivers to enable them to continue with their role during pandemic.

## Introduction

Italy was the first European country declaring in March 2020 a national lockdown to contain contagion after pandemic of the new Coronavirus SARS-Cov2, forcing the population into social restrictions, with individuals being allowed to leave home only for limited and recognized necessities. The experience of lockdown is often unpleasant for different level of stressors as social separation, loss of freedom, perception of uncertainty and boredom, with negative psychological effects including post-traumatic stress symptoms, confusion, and anger in normal population (1). Evidence of lockdown from previous viral outbreaks reported a substantial impact on general mental health, as anxiety, mood alterations and post-traumatic stress symptoms (2–4). During COVID-19 pandemic, post-traumatic stress symptoms (37, 1%), depressive symptoms (17, 3%), anxiety (20, 8%) (5), changes in appetite, reduced libido (6), and altered sleeping patterns (6, 7) were revealed in the general population. Among stressors associated with negative psychological impact during lockdown, female gender (5, 6, 8), lower levels of education (8), story of previous psychiatric illness, and being a care worker (1) were most relevant.

Elderly people with cognitive decline and their caregivers had negative outcomes from the experience of social isolation imposed by pandemic. Even in non-pandemic context, family members caring for individuals with dementia often describe this experience as “enduring stress and frustration” and the term caregiver burden is most often used to describe this phenomenon (9). Indeed, the contextual experience of caring a person with dementia lead to psychologically stressful and physically exhausting tasks, with significant expenditure of time, energy, and money over potentially long periods of time (10, 11).

During pandemic, direct outcomes of restrictive measures induces a substantial change in patients with dementia and caregivers' daily routine, with reductions of physical and cognitive activities change in lifestyle and in the management of the disease (12, 13). Increment of behavioral disturbances as agitation, sleep disorders, hallucinations, wandering, anxiety, apathy, and depression occurred in patients with dementia (14–16). Caregiver distress and psychological well-being exacerbated (17), possibly acting in a vicious loop with mutual increase of psychiatric burden (15) and caregiver burden (18, 19). These significant social stressors, together with worsening of patient's cognitive, behavioral and motor deficits (14, 15), increased caregiver stress perception during COVID-19 pandemic (17).

Studies during the first lockdown in Italy investigated caregiver stress perception with telephone surveys, demonstrating the presence of stress-related symptoms reported by 65,9% of caregivers including anxiety, helplessness, anguish, irritability, abandonment, and feelings of depression (14, 15). Other studies in Italy (17, 19, 20) and Spain (12, 16) had similar results, all highlighting that lockdown led to warning psychological response in caregiver of patients with dementia during the first months of pandemic. After the first lockdown in March 2020, a new decree-law in force since October 8 confirmed the previous containment measures, and from November 6 containment for differentiated scenarios and curfew were established. Lockdown periods, partial limitations of social life and worries of contamination due to ongoing pandemic endured until a vaccination campaign was started at the beginning of 2021.

To our knowledge, no studies are available assessing long-term psychological effects of pandemic in caregivers of persons with dementia. The aim of this study was to longitudinally investigate the long-term psychological impact of restrictive measures in caregivers of patients with dementia assessed in March 2020, at the beginning of social isolation due to COVID-19 pandemic, and 1 year later (March 2021). In details, presence and severity of depression, anxiety, change of sleep quality, caregiver burden and coping strategies after 1 year of pandemic will be assessed, and their demographic, social and psychological predictors at the beginning of lockdown will be evaluated.

## Methods

### Participants

A total of 151 individuals, 101 informal caregivers of persons with dementia and 50 subjects without caregiving duties were enrolled in this study.

Caregivers were all family members or careers of patients with cognitive decline regularly attending the Memory Clinic of the Neurology Unit at the Hospital of Padua. Of the 101 caregivers assessed at baseline in March 2020 (T1), 16 caregivers were not assessed at follow-up (T2) performed in March 2021 and therefore the final sample of informal caregivers considered for statistical analyses consisted of 85 subjects with both T1 and T2 evaluations. Reasons for drop out from the longitudinal study were: withdrawn of consent (*n* = 6), loss of contacts (*n* = 8), institutionalization or death of their care receiver (*n* = 2). Fifty non-caregivers subjects were evaluated only in March 2021. Non-caregivers were selected from volunteers of charities and social services or referred from general practitioners. Inclusion criteria were: living at home in the last year; functional independency; age within in the range similar to that of caregivers. Exclusion criteria were: presence of neurological or psychiatric disorders; caring for a person with physical or mental disability; having had COVID infection with hospitalization in the last year. Psychiatric and psychopathological history was screened before starting the phone interview with a short anamnestic questionnaire investigating possible neurological (i.e., Parkinson's disease, stroke, epilepsy) or psychiatric (i.e., anxiety, depression) disorders and/or the use of psychotropic drugs.

None of the subjects involved in the study was affected by COVID-19 infection in the period of observation.

## Materials and Methods

This is a longitudinal study with 1-year follow-up and two-time points assessments. At baseline participants were interviewed during the first COVID-19 lockdown in March 2020 (T1) assessing socio-demographic characteristics of the caregiver, continuity of therapeutic care, self-perception of distress symptoms such as anxiety, insomnia, irritability, and specific variables of wellbeing such as working conditions, cohabitation, and social support. Methodology of this phone-based interview has been already published (14). After one-year from baseline a second follow-up assessment (T2) was performed through a telephone interview administrated to all participants by the same experimenter (TB) between the 22^nd^ March and 24^th^ April 2021. The follow-up assessment consisted of two parts: (1) a semi-structure interview using the same questionnaires administered at T1 enquiring on changes of socio-demographic characteristics, self-perception of distress symptoms, global health and COVID-19 infection; (2) standardized questionnaires and scales to assess presence and severity of depression, anxiety, sleep changes, caregiver burden and coping abilities. The degree of depression and anxiety was assessed with the Depression, Anxiety and Stress Scale (DASS-21); sleep quality with the Pittsburgh Sleep Quality Index (PSQI), the caregiver burden with the Caregiver Burden Inventory (CBI) and coping strategies with the Coping Orientation to Problems Experienced – Nuova Versione Italiana (COPE-NVI).

DASS-21 (21, 22) was used for the assessment of depression (dysphoria, hopelessness, devaluation of life, lack of interests and incentive, low self-esteem, and anhedonia), and anxiety (somatic and subjective symptoms of anxiety, autonomic arousal, and situational anxiety) symptoms. Each subscale consists of seven items rated on a 4-points Likert scale (from 0 “*never*” to 3 “*nearly always*”). The total score for each sub-scale is given by the sum of its items multiplied by two, where higher values correspond to a higher and more severe alteration. Recommended cut-off scores for severity levels were considered as following: depression = normal 0–9, mild 10–13; moderate 14–20, severe >21; anxiety = normal 0–7, mild 8–9; moderate 10–14, severe >15 (23).

A modified version of PSQI (23, 24) was used for assessing the following four domains of sleep/wake disorders: (1). Sleep quality subjective perception; (2); Habitual sleep efficiency (including sleep duration as sleep time/bedtime). The range of scores for both variables is between 0 and 3 for, respectively “very bad” and “very good”; (3). Use of sleeping medications and 4; Daytime dysfunctions (i.e., daytime sleepiness, or lack of energies during the day). The range of scores for these two last variables is between 0 and 3, where 0 indicates low frequencies, as “not during the past month” and 3 indicates high frequencies (“more than 3 times a week”).

CBI (25, 26) explores five burden domains: time-dependence burden, developmental burden, physical burden, social burden, and emotional burden. Due to time-dependent constrains of a telehone interview, from CBI we chose to explore the following three domains: (1). “Time-dependence burden,” due to time and energy spent for the constant vigilance and sense of responsibility – “*My care receiver is dependent on me*” or “*I don't have a minute's break from my caregiving chores*“; (2). “Developmental burden,” relying to the sense of failure in development and guilty with respect to peers due to failure of role expectations of becoming caregiver - items like “*Why did this happen to me*?” or “*I expected that things would be different at this point in my life*” and (3). “Emotional burden,” describing caregivers' negative feelings toward care receivers, which may result from the patient's unpredictable and often bizarre behavior - items like, “*I resent my care receiver*” and “*I feel angry about my interactions with my care receiver*.” Each factor consisted of five items rated on a 5-points Likert scale (from 0 “never” to 4 “nearly always”). The social burden and physical burden were not investigated considering the confounding variables introduced during pandemic as far as social restrictions and physical symptoms.

COPE-NVI (27) was administered to assess the ability to manage traumatic events or stressful situations. In particular, we investigated four dimensions: (1). Social support (tendency to seek understanding, support and information from others); (2). Avoidance strategies (tendency to use behavioral and mental denial and detachment); (3). Positive attitude (tendency to adopt a positive acceptance and reinterpretation of events) and (4). Problem orientation (tendency to use active planning strategies). Each domain is rated on a 4-points Likert scale from 1 “*usually I did not do it*” to 4 “I almost always do it.” The total score for each scale is given by the sum of its items, where higher values refer to a greater tendency to use that specific coping strategy.

Non-caregivers controls were assessed only at T2 with DASS-21, PSQI and COPE-NVI questionnaires. All the questionnaires were administered with the same chronological order in the two groups.

All participants were asked for a prior consent, guaranteeing them total anonymity in the processing of data.

### Statistical Analysis

Answers to the telephonic survey were first analyzed using frequency analysis to assess differences and longitudinal changes (i.e., T1 vs. T2) in caregivers.

Then, long-term psychological effects of pandemic were analyzed by comparing caregivers and non-caregivers across a series of scales administered in T2. Notably, despite significant differences between caregivers and non-caregivers in the education level, we did not use education as covariate since its impact on DASS-21, PSQI and COPE-NVI scores has been shown negligible (22, 23, 27).

Specifically, we investigated the impact of being a caregiver (i.e., Factor 1) by comparing caregivers and non-caregivers, and the impact of being in a pandemic (i.e., Factor 2) by comparing non-caregivers and normative data, which were collected from non-caregivers and not during a pandemic. Finally, the comparison between caregivers and normative data allowed us to study the cumulative effect of such factors (i.e., the impact of being a caregiver during a pandemic). Bonferroni correction for multiple comparison was applied.

Notably, in all comparisons t-tests were used after checking the reliability of the results also with non-parametric statistics (i.e., Mann-Whitney test).

Finally, we aimed to find baseline predictors of worse psychological outcome in T2. To this end, we first compared *via t*-test the level of DASS-21-Depression scores (T2) of caregivers who answered “Yes” or “No” to the first survey items (T1) regarding the presence of insomnia (sleep disturbances), depression and anxiety. Then, we built a series of stepwise linear regression models based on demographic features (i.e., caregiver's sex, age and education, patient's age and sex) and T1 survey responses to predict T2 more relevant psychological scores (PSQI-Sleep Quality, DASS-21-Depression and DASS-21-Anxiety), which were checked for normality of residuals by means of the Shapiro-Wilk test.

The prediction was realized with a leave-one-out cross-validation (LOOCV) design and its accuracy was evaluated as the correlation between actual and predicted score values. All analyses were ran using R software version 3.6.2.

## Results

### Socio-Demographic Characteristics

Caregivers and non-caregivers controls were matched for age (caregivers: mean = 62 ± 14,6; non-caregivers: M = 62 ± 11,2 years; *p* = 0.869) and gender (female, caregivers: 69,4%, *n* = 59; non-caregivers: 64%, *n* = 32; *p* = 0.492) while education level was higher for non-caregivers (caregivers: mean = 11,6 ± 4,3; non-caregivers: mean = 13,06 ± 3,9 years; *p* = 0.023). The majority of caregivers were spouses (57,6%, n = 49) and cohabitant with the patient (67,1%, *n* = 57). Patients cared by caregivers recruited in the study (mean age 74,62 ± 11,3) were affected by dementia with different etiology: 51 with Alzheimer's Disease, 26 with dementia with Lewy Body, 6 with Frontotemporal Dementia, 2 with Vascular Dementia. Severity of cognitive impairment measured with the Clinician Dementia Rating (CDR) scale was mild in the majority of patients having CDR 1 in 60% (*n* = 51) of cases, CDR 2 (moderate stage) in 19% (*n* = 16) and CDR 3 (severe) in 21% (*n* = 18).

At T2, 81,2% (*n* = 69) of caregivers did not change work status during pandemic while 18,8% (*n* = 16) did it, such as implementation of remote working (9,4%, *n* = 8), loss of employment (4,7%, *n* = 4) and increase in the amount of work (4,7%, *n* = 4). Difficulties with the continuity of care were reported more frequently at T2 than T1 (38,8 vs.17,7%; *p* = 0.006). 15% (*n* = 13) needed the help of a formal caregiving.

### Change of Frequency of Self-Reported Stress Related Symptoms

After 1 year of pandemic almost all caregivers (96,5%, *n* = 82) reported at least one stress-related symptom. Depression and perception of sadness were the most prevalent complaints, been reported by 60% (*n* = 51) of participants, followed by feelings of irritability (49,4%, *n* = 42), anxiety (45,9%, *n* = 39), insomnia (40%, *n* = 34), isolation (41,2%, *n* = 35), overwhelm (37,6%, *n* = 32), anguish (31,8%, *n* = 27) and abandonment (25,9%, *n* = 22). The 37,6% (*n* = 32) of the caregivers reported feeling of calmness.

**Figure 1:**
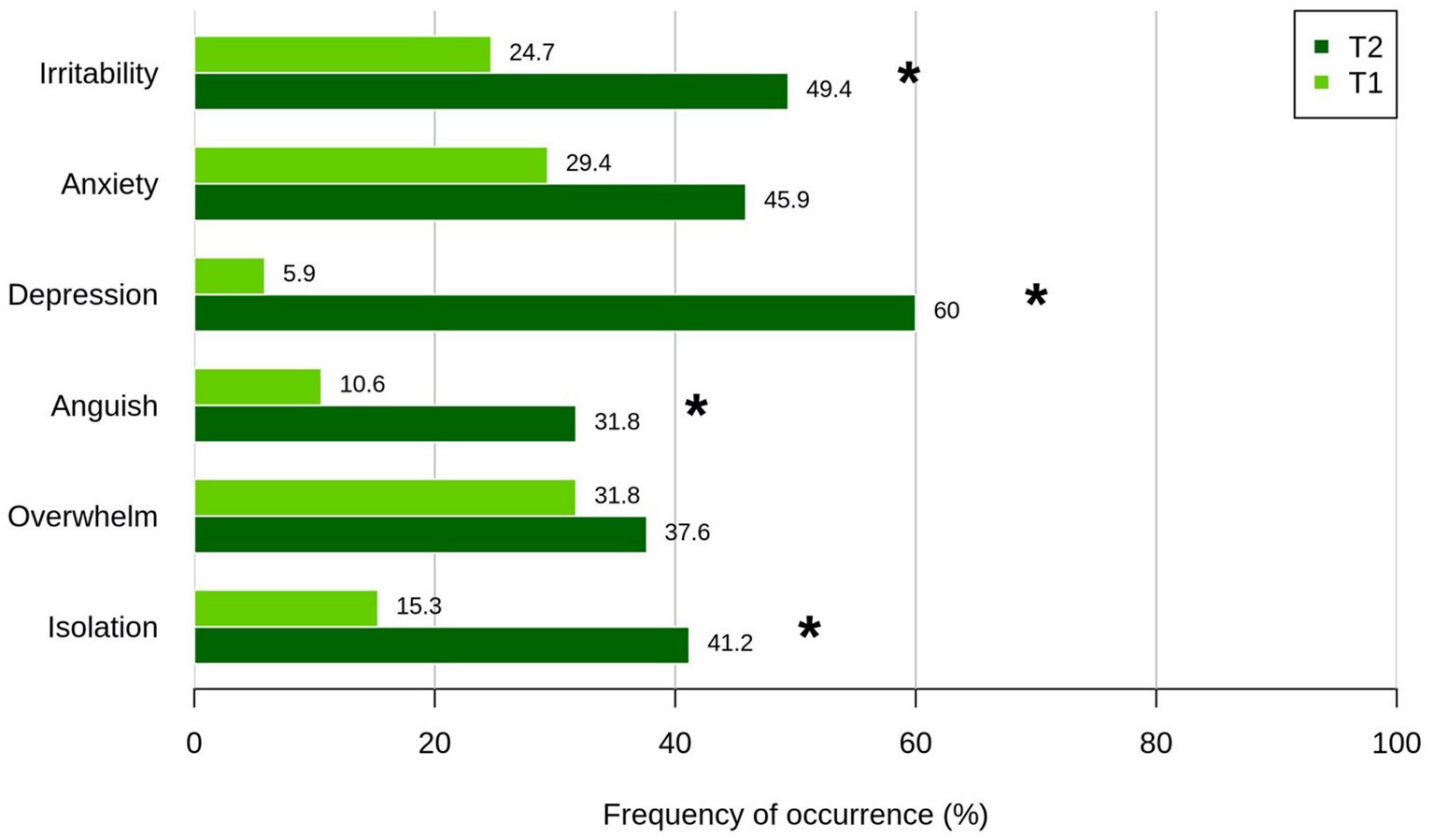
Frequency of stress-related symptoms reported by caregivers during the first lockdown (T1, March 2020; light green) and 1 year after first restrictions (T2, March-April 2021, dark green). T1 and T2 proportion of answers were compared by means of a series of McNemar's χ^2^ tests for paired comparisons. *Bonferroni-corrected significant difference.

The frequency of stress-related symptoms was increased at T2 respect to T1 (Figure 1). A significant difference surviving Bonferroni correction between T2 and T1 was found particularly for depression (χ^2^(1) = 42,188, *p* < 0.001), isolation (χ^2^(1) = 14,7; *p* < 0.001), irritability (χ^2^(1) = 11,429; *p* < 0.001), and anguish (χ^2^(1) = 11,115, *p* < 0.001). Anxiety was reported more frequently at T2 (45,9%) than T1 (29,4%) but difference did not survive multiple comparisons correction (χ^2^(1) = 4,46; uncorrected *p* = 0.0.35).

### Psychological Long-Term Effects of Social Restrictions

**Figure 2:**
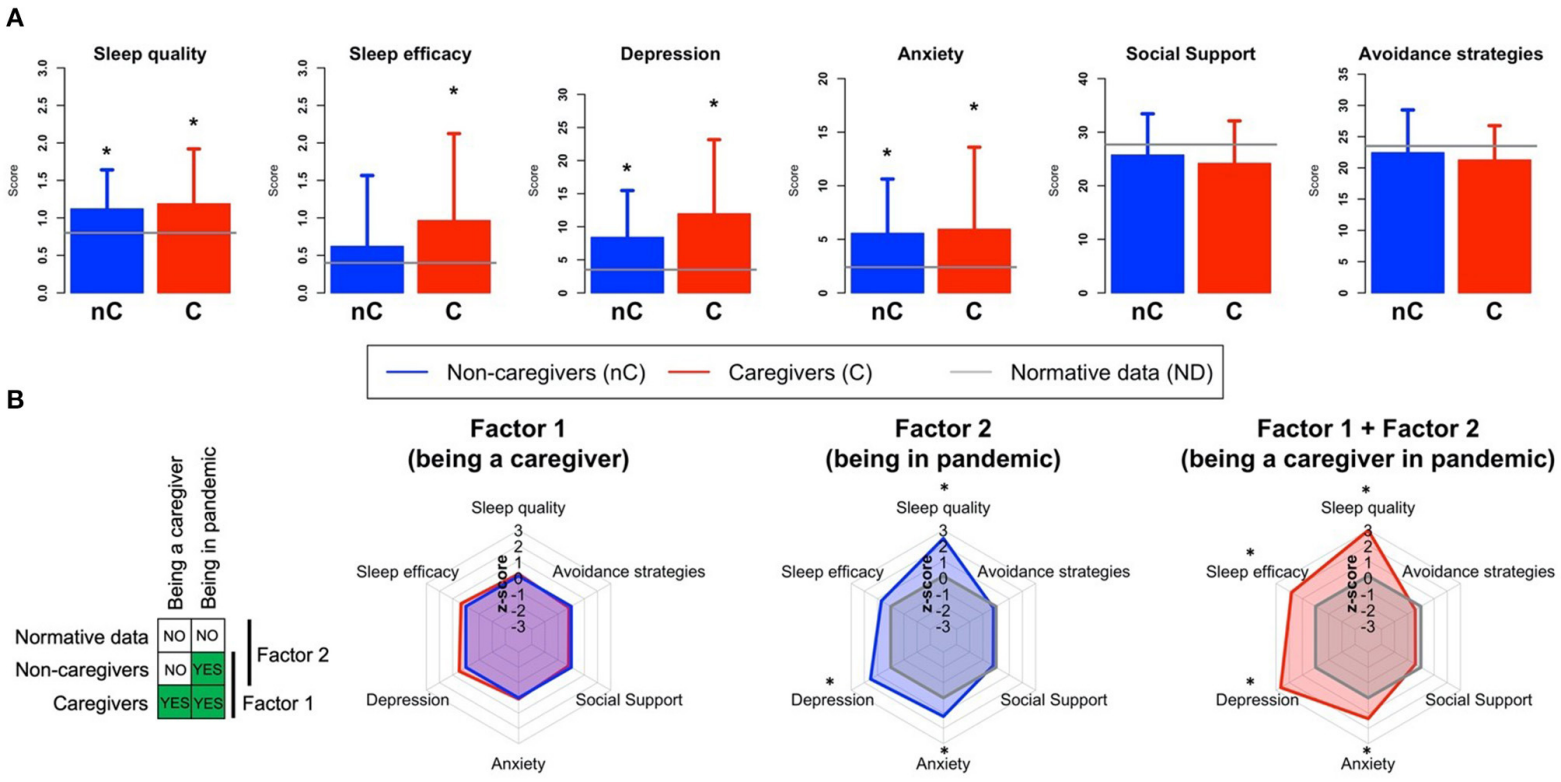
**(A)** Comparisons between caregivers (C), non-caregivers (nC) for PSQI-Sleep Quality Perception, PSQI-Sleep Efficiency, DASS-Depression, DASS-Anxiety, COPE-NVI-Social Support and COPE-NVI-Avoidance Strategies scores. Dashed lines indicated normative data (ND) level for each component. Higher values for the PSQI quality perception and efficiency scores mean worse performance (bad quality perception and bad habitual efficiency). *significant difference with the normative data. **(B)** Radar plots show the results of the investigation of the role of different components of caregivers' stress on depression, anxiety, sleep abnormalities and coping strategies in T2. Factor 1 (being a caregiver) was analyzed by comparing caregivers and non-caregivers (both in pandemic). Factor 2 (being in a pandemic) was investigated by comparing non-caregivers with normative data (i.e., not collected during a pandemic). Finally, we investigated both Factor 1 and Factor 2 (being a caregiver in pandemic) by comparing caregivers and normative data. The radar plots show the comparison between two groups, with one group used as reference (i.e., the scores of one group were z-scored on the reference group). For Factor 1 the non-caregivers were the reference group, for Factor 2 and 3 we used the normative data as reference.

In Table 1 and Figure 2A are shown results obtained from the comparison of mean scores from scales assessing depression, anxiety, sleep abnormalities and coping strategies in caregivers and non-caregivers groups at T2.

**Table 1:** Descriptive measures (mean ± SD) of each questionnaires' component with caregivers and non-caregivers' comparisons. Descriptive measures (mean ± SD) for each component were shown by normative data (22, 23, 26).

**Variables assessed at T2**	**Caregivers (*n* = 85)**	**Non-caregivers (*n* = 50)**	***P (uncorrected.)***	***Normative data***
	***Mean ±SD***	***Mean ±SD***		
DASS-21-depression	11.96 ± 11.19	8.40 ± 7.07	0.025	3.5 ± 3.2
DASS-21–anxiety	5.94 ± 7.66	5.56 ± 5.07	0.73	2.4 ± 2.6
PSQI–sleep quality	1.19 ± 0.73	1.12 ± 0.52	0.53	0.80 ± 0.13
PSQI–habitudinal sleep efficiency	0.96 ± 1.16	0.62 ± 0.94	0.23	0.40 ± 0.31
PSQI–use of sleeping medication	0.76 ±1.29	0.24 ± 0.74	0.003	0.10 ± 0.10
PSQI–daytime dysfunction	0.60 ± 0.83	0.44 ± 0.64	0.22	0.60 ± 0.27
CBI–time dependence burden	10.84 ± 6.49	-	-	6.98 ± 5.89
CBI–developmental burden	7.94 ± 6.28	-	-	7.08 ± 5.89
CBI–emotional burden	3.04 ± 3.07	-	-	2.02 ± 3.04
COPE-NVI–social support	24.18 ± 7.94	25.74 ± 7.69	0.26	27.7 ± 8.4
COPE-NVI–avoidance strategies	21.27 ± 5.54	22.44 ± 6.83	0.31	23.5 ± 5.1
COPE-NVI–positive attitude	31.13 ± 8.04	32.96 ± 8.86	0.23	30.9 ± 6
COPE-NVI–problem orientation	32.84 ± 8.15	33.82 ± 8.44	0.51	32 ± 6.7

*DASS-21, Depression, Anxiety and Stress Scale; PSQI, Pittsburgh Sleep Quality Index; CBI, Caregiver Burden Inventory; COPE-NVI, Coping Orientation to the Problem Experienced-Nuova Versione Italiana*.

Both caregivers and non-caregivers showed higher mean scores compared to non-pandemic normative data in the DASS-21-Depression and Anxiety scale (23) and worse sleep disturbances scores at the Sleep Quality Perception and Habitual Sleep Efficacy subitems of PSQI questionnaire (26). Higher levels of depression and more frequent use of sleep inducers were detected in caregivers compared to non-caregivers although differences did not survive multiple comparisons correction. Regarding COPE-NVI performances, caregivers showed lower scores in all coping dimensions. Taken together, these results suggest that a common feature shared by both caregivers and non-caregivers played a main role as stressor.

In the attempt to disentangle the relative contribution of different stressors, we identified two factors, namely being caregiver (Factor 1) and living in a pandemic situation (Factor 2). To this end, we selectively compared by means of a series of *t*-tests caregivers and non-caregivers both each other and with normative data, across the DASS-21, PSQI and COPE-NVI scales. Specifically, the role of Factor 1 was investigated by comparing caregivers and non-caregivers, while Factor 2 was highlighted by comparing non-caregivers with normative data (assuming that the normative sample for all scales was composed by individuals without caregiving duties and not dealing with a pandemic). Moreover, we evaluated the possible cumulative effect of both factors on stress symptoms (i.e., Factor 1 + Factor 2 = being a caregiver in a pandemic). The results of this analysis are reported in (Figure 2B), and showed that being a caregiver (Factor 1) was not the main driver of stress symptoms. Indeed, caregivers and non-caregivers scores were highly similar. On the other hand, living in a pandemic (Factor 2) contributed to increase anxiety, depression and sleep abnormalities (*p* < 0.001), and the combination of the two factors (being a caregiver in pandemic) showed a cumulative effect on the same symptoms as well as on sleep efficacy (*p* < 0.001). This suggests that being a caregiver further increased psychological outcomes of the pandemic especially regarding of sleep changes.

**Figure 3:**
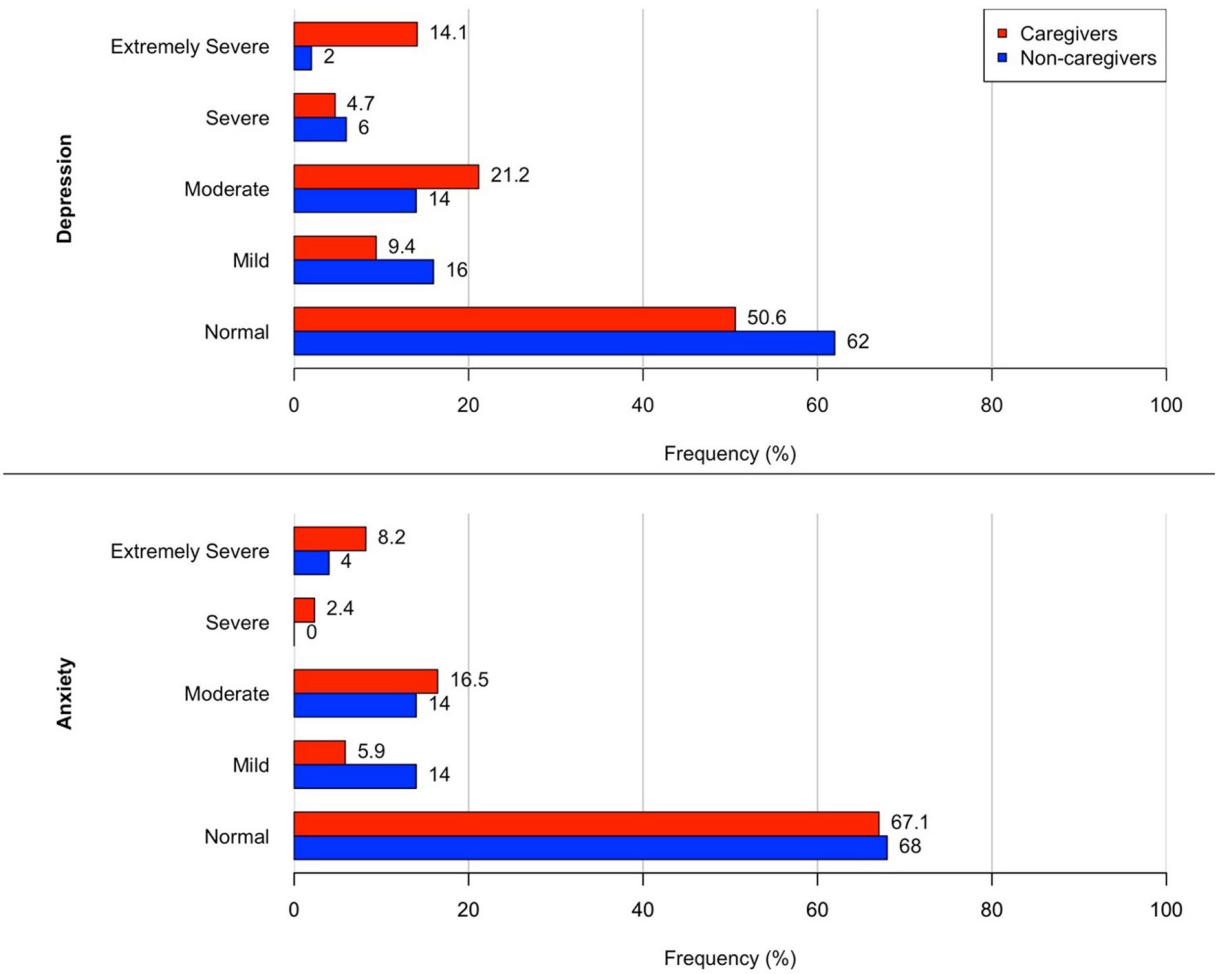
Frequency distribution of DASS-21 Anxiety and Depression levels of severity in caregivers (red) and non-caregivers (blue) 1 year after first restrictions (T2, March-April 2021), Recommended cut-off scores for severity levels were considered as following: depression = normal 0–9, mild 10–13, moderate 14–20, severe > 21; anxiety = normal 0–7, mild 8–9, moderate 10–14, severe > 15 (23).

Forty-nine percent of caregivers (*n* = 42) had DASS-21-Depression scores above the cut-off levels for normality (>9) compared to 38% (*n* = 19) of non-caregivers, having more frequently extremely severe levels of depression compared to non-caregivers (χ^2^(1) = 9.11, *p* = 0.0025) (Figure 3). Regarding DASS-21-Anxiety, 32% (*n* = 27) of caregivers had scores above normal cut-off levels (>7) respect to 30% (*n* = 15) of non-caregivers. Caregivers had prevalent moderate and severe degrees of anxiety and non-caregivers had prevalent mild levels of anxiety (Figure 3).

Caregiver burden was higher during pandemic respect to normative values in the non-pandemic scenario (21) in all the three CBI-domains investigated, especially for time-dependence burden.

Results from correlation analysis between the variables detected at T2 for each group (caregivers and non-caregivers) are shown in (Supplementary Figure 1).

Within the caregivers' group, higher DASS-21 Depression scores correlated with higher CBI-developmental burden (r(85) = 0.66, *p* < 0.05) and emotional burden (r(85) = 0.57, *p* < 0.05). Depression levels were positively associated with higher levels of avoidance coping strategies both in caregivers (r(85) = 0.62 *p* < 0.05) and in non-caregivers (r(85) = 0.48 *p* < 0.05). Only in the caregivers' group higher depression scores correlated with higher anxiety (r(85) = 0.55, *p* < 0.05).

### Baseline Predictors of Long-Term Psychological Outcomes

**Figure 4:**
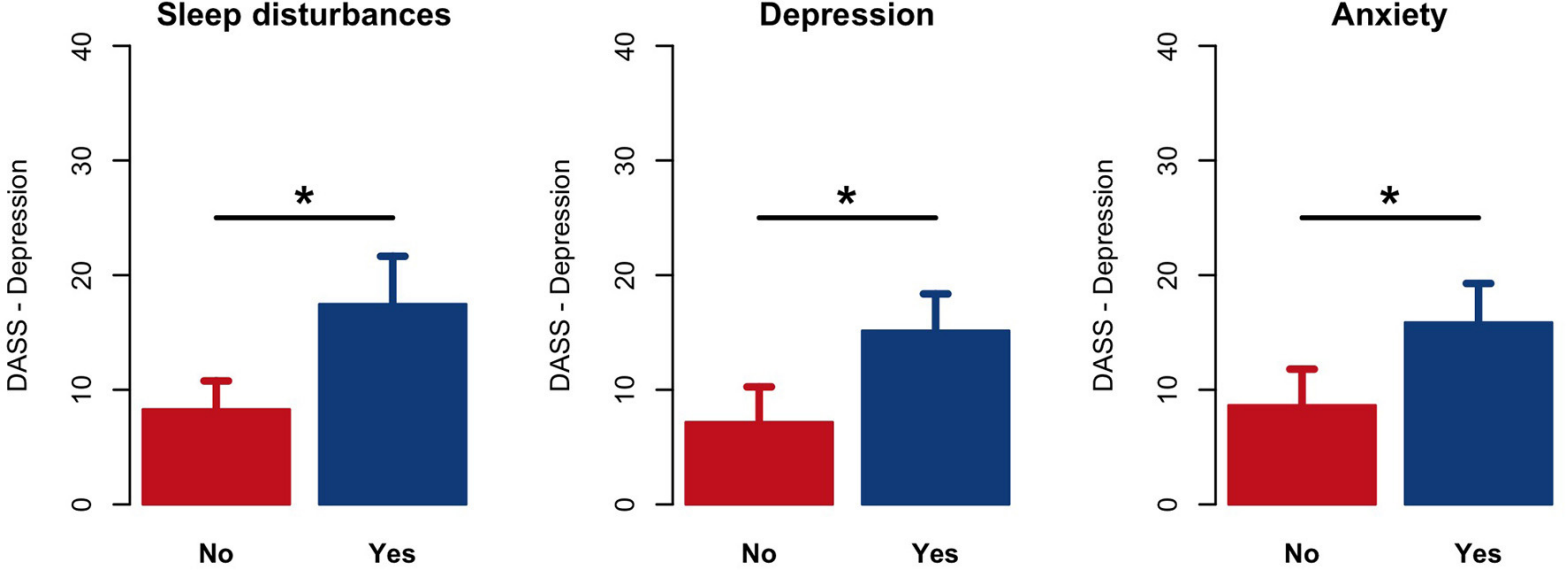
Comparison results of DASS-21-Depression scores at T2 between those caregivers who answered “Yes” or “No” to the first survey items (T1) regarding the presence of insomnia (sleep disturbances), depression and anxiety. *p < 0.001.

Self-reported perception of insomnia, depression and anxiety at T1 were significantly associated with DASS-depression symptoms at T2 (*p* < 0.001) (Figure 4).

We built a series of linear regression models from T1 variables to predict depression, sleep quality and anxiety at T2. Specifically, one model was built for each variable with the aim of highlighting the best set of T1 predictors of T2 symptoms.

The model best predicting depression at T2 included female sex (*p* < 0.001) low education level (*p* = 0.04), feelings of being overwhelm (*p* = 0.02) and isolated (*p* = 0.04) among all demographic, socio-relational, psychological, and COVID-19 care related variables selected from the baseline survey (*p* < 0.001) (Table 2).

**Table 2:** Variables included in the predictive model for depression, sleep quality and anxiety levels at T2.

**Predictors**	**Depression**				**Sleep quality**				**Anxiety**			
	***Beta***	***SE***	***t***	***p***	***Beta***	***SE***	***t***	***p***	***Beta***	***SE***	***t***	***p***
Sex (caregiver)	−0.19	0.05	−3.61	<0.001	−0.19	0.05	−3.61	<0.001	−0.11	0.05	−2.17	0.03
Education (caregiver)	−0.01	0.01	−2.15	0.04	−0.01	0.01	−2.15	0.04	−0.01	0.01	−2.39	0.02
Isolation	0.17	0.08	2.12	0.04	0.17	0.08	2.12	0.04	-	-	-	-
Overwhelm	0.14	0.06	2.29	0.02	0.14	0.06	2.29	0.02	0.11	0.05	2.16	0.03
Distress	0.13	0.08	1.53	0.13	0.13	0.08	1.53	0.13	-	-	-	-
Care continuity	−0.1	0.06	−1.66	0.1	−0.1	0.06	−1.66	0.1	-	-	-	-
Irritability	-	-	-	-	-	-	-	-	0.18	0.06	2.94	<0.001
Life change–time	-	-	-	-	-	-	-	-	0.23	0.07	3.46	<0.001
Other people	-	-	-	-	-	-	-	-	0.13	0.05	2.79	0.01
Relation change–worse	-	-	-	-	-	-	-	-	−0.19	0.09	−2.14	0.04
Social support	-	-	-	-	-	-	-	-	0.09	0.05	1.99	0.05
Life change–relation	-	-	-	-	-	-	-	-	−0.15	0.09	−1.62	0.11

*Predictive power of the model for depression F[6,78] = 8.54, p < 0.001, R^2^ = 0.35; sleep quality: F[9,75] = 4.94, p < 0.001, R^2^ = 0.29; and anxiety: F[6,78] = 1.45, p < 0.001, R^2^ = 0.24*.

Female sex was also predictive of worse sleep quality (*p* = 0.01) and higher anxiety (*p* = 0.03). Several psychological, burden-related and social variables explained anxiety 1 year after the first restrictions (Table 2).

Finally, we used the models described above to predict depression, anxiety and sleep quality scores at T2 with a Leave-One-Out Cross-Validation (LOOCV) design. The results showed that the model-predicted scores significantly correlated with actual scores in all cases (all *p* < 0.001), and again, the strongest correlation (i.e., prediction accuracy) observed was for depression (r = 0.53, *p* < 0.001) (Supplementary Figure 2).

## Discussion

Self-perceived of psychological stress-related symptoms were significantly increased after 1 year of COVID-19 pandemic in comparison with the frequency detected in the first lockdown phase. Ninety-six percent of caregivers reported at least one stress-related symptom, with depression being the most frequently reported 1 year after the beginning of pandemic, followed by irritability, anxiety, and sleep alterations. *Ad-hoc* scales showed increased levels of depression and worse sleep quality in both caregivers and non-caregivers, with higher frequency of severe degree of depression in caregivers. Increased levels of depressive symptoms were predicted by female sex, lower education, perception of isolation, and overwhelm at the beginning of pandemic.

### Psychological Outcomes After 1 Year of Pandemic

The large prevalence of stress in family caregivers of patients with dementia has been observed during the first restrictions due to COVID-19 in Italy (14, 15, 17). This study highlighted evidence of a long-term psychological impact of isolation in dementia caregivers, with further increment of stress-related symptoms, particularly depression.

While only 5,9% of caregivers reported feelings of depression at the first lockdown in March 2020, the frequency of subjective depressive symptoms highly increased to 60% at 1-year follow-up. Self-perception of having depressive symptoms was confirmed by higher scores at DASS-Depression scale detected in almost 50% of caregivers (24). Increased levels of depression were also detected in 38% of persons not involved in caregiving, highlighting pandemic as a common stressor in the general population. During the first lockdown increased levels of depression were found in the general populations (6, 28, 29). Mild/severe levels of depression increased up to 32,3% in the last week of restrictions in Italy, compared to 15,4% before lockdown (6). Both caregivers and non-caregivers had also higher scores at the DASS-21-Anxiety at T2 compared to non-pandemic normative data without significant difference between the two groups.

Frequency distribution of self-perception of stress-related psychological symptoms changed from acute to long-term stage of pandemic. After 1-year of social isolation due to pandemic, depressive symptoms were more frequent than anxiety symptoms. This observation could be interpreted as persisting effects of a stressful situation. According to the General Adaptation Syndrome (30), the human reaction to an extreme stress situation occurs through three different stages. Firstly, the alarm reaction stage refers to the initial symptoms experienced in a stressful condition and involves a “fight-or-flight” response. This is a physiological response to stress that prepares the system either to flee or to protect itself in dangerous situations. Secondly, in the resistance stage the subject tries to manage and adapt to the negative effects of the prolonged stress. Finally, when the stress factors become chronic, the exhaustion takes place. Considering the COVID-19 pandemic a chronic stressor, after a first phase of alarm with a greater prevalence in caregivers of perceived anxiety, the exposure to prolonged uncertainty may have induced a phase of exhaustion with higher prevalence of depressive symptoms respect to the first phase of alarm with greater prevalence of perceived anxiety.

Caregivers and non-caregivers showed worse sleep quality perception and sleep efficacy than in normal conditions (25). Although caregivers had worse sleep quality than non-caregivers, differences did not survive correction for multiple comparison. Prior non-pandemic literature showed that dementia caregivers had poorer perceived sleep quality and shorter sleep duration than non-caregivers (31). Cellini and colleagues (7) showed that sleep-wake rhythms markedly changed in the Italian population during the first lockdown, with people spending more time in bed, but also reporting a lower sleep quality (7). Alteration in sleep quality and sleep efficacy was associated with an increased feeling of expansion of time. Several studies (32–35) during the first lockdown showed that one of the main consequences of restrictive measures was an alteration in people's relationship to time. Time seemed to pass far more slowly compared to before the lockdown. Moreover, since people suffering from depression already found that time passes slower than other people (36), lockdown could have increased this perception even more.

### Baseline Predictors of Long-Term Psychological Outcomes

Caregivers' stress responses are mediated by a variety of factors relating to both socio-demographic and psychological variables such as gender, kinship ties (9), self-efficacy and coping strategies (37). In our study depression, poor sleep quality and anxiety were predicted by lower education and female sex. Being a female caregiver is considered an important risk factor for health problems, depressive symptoms and caregiver burden (9, 38). Being female is also a risk factor of negative psychological impact during COVID-19 lockdown (5, 6). Women could get overloaded with additional family, household and working activities under restrictions (6).

Among psychological variables, presence of feelings of overwhelm at the beginning of pandemic was the best predictors of long-term depression and anxiety, while feelings of isolation and sleep alterations were predictors of subsequent depression. Increased anxiety was predicted by changes regarding socio-relational variables, such as the amount of time needed for assistance, decreased quality in the relationship with the care-receiver, need of support. These predictive factors, which affect mood and sleep over the long term, should be taken in consideration for prevention strategies in caregivers of people with dementia.

### Caregivers Burden and Coping Strategies

Caregivers had higher levels in time dependence burden, developmental/psychological burden and emotional burden during the COVID-19 pandemic, indicating that the restrictive measures might have charged caregivers with additional challenges and responsibilities affecting the caregiver burden in normal condition (18, 39).

Higher levels of developmental/psychological burden and emotional burden were positively associated with higher depression. The significant relationship between developmental burden and depression is consistently reported in literature (40, 41). The developmental/psychological dimension of caregiver burden is associated with depression to a greater extent than other types of caregiver burden (42), and caregivers who feel deprived of doing things were more likely prone to depression (41). Regarding the emotional burden, past studies failed to find significant associations with depression (40, 41).

It is known that subjects tend to respond to increasing burden and stressful events with three different types of internal coping strategies: emotion-focused strategies (acceptance, emotional support, humor, positive reframing, and religion), problem-focused strategies (active coping, instrumental support, and planning) and dysfunctional coping (behavioural disengagement, denial, self-distraction, self-blame, substance use) (43). Within the caregivers' group, increased levels of depression and anxiety were closely related with dysfunctional coping strategies in pandemic. These findings are consistent with previous research in caregivers underlying an association between stress-coping strategy focused on avoidance and levels of depression, anxiety, and burden (44–48). Avoidance coping can be useful during the first phase of an uncontrollable stressful situation, as protective and defensive strategy. Yet, with the chronicity of the stressful situation, this strategy is less used with increasing risk of depression (22, 49).

Social support represents another external component of coping (50). Caregivers tend to use less social support strategies and avoidance strategies (22). Social support is often negatively correlated with depressive symptoms in elderly, and in caregivers of patients with Alzheimer's disease (51). Furthermore, despite social support is generally considered a protective factor for depression (52), female caregivers usually tend to refuse other's help, isolating themselves (53), with a possible increasing of subjective burden perception (9).

Our results indicate that maintaining lower social support due to persistence of restrictions and dysfunctional coping strategies are risk factor for developing of depressive symptoms in caregivers of persons with dementia.

### Study Limitations

There are a few limitations of this study to be considered. First, the study started during the acute phase of pandemic and collection of data using *ad-hoc* scales assessing behavioral features such as depression, anxiety, sleep alteration, and coping strategies of a stressful events was not feasible. Therefore, these measures were only available at follow-up time-point and direct comparison between baseline and follow-up assessments was not allowed. Nonetheless, we could analyze other baseline variables obtained within a large survey performed in the acute pandemic stage allowing to investigate predictive demographic, social and psychological features of worse mental health outcomes in caregivers. Secondly, we did not assess correlations between severity of patients' behavioral and psychological burden and symptoms of caregivers distress. Finally, although the present study did not acquire information about caregivers' mental state before the pandemic outbreak, the administration of the interview in two different periods during pandemic allowed to track changes of psychological well-being during a chronic stressor in add-on to the burden due to caregiving.

## Conclusion

In accordance with some recent studies (6, 7, 14, 15, 19), we confirm long-term psychological stress-related symptoms including depression, anxiety, and sleep quality disorders in caregivers of persons with dementia during COVID-19 restrictions in Italy. Self-perceptions of higher levels of depression, anxiety and poor sleep quality and sleep efficacy were confirmed by standardized questionnaires. Depression was more frequent after 1 year of pandemic that at the beginning of lockdown suggesting that chronic exposure to stressful events may have led to exhaustion of psychological resources. Female caregivers and those with lower education have the higher risk of depression.

Providing information about effective coping strategies together with “how to cope” may be useful to deal and cope with emergent issues (54). To this purpose, a combined support intervention targeting multiple levels of the stress/health model could produce a significant improvement in both caregiver burden and wellbeing, focusing on reducing caregivers' loneliness and on psychoeducational interventions to relieve anxiety, enhance awareness and healthy behaviors and reduce family conflicts, promoting the active listening, and mutual support between family members (55).

## Data Availability Statement

The original contributions presented in the study are included in the article/Supplementary Material, further inquiries can be directed to the corresponding author.

## Ethics Statement

The studies involving human participants were reviewed and approved by Ethics Committee of the Azienda Ospedaliera of Padova (Italy). The patients/participants provided their written informed consent to participate in this study.

## Author Contributions

CB: designed the study and wrote the manuscript. TB: collected data and wrote the draft. MZ: revised the draft. IR: supervised the study and revised the draft. SM: collected the data. AZ: analyzed the data and revised the draft. AC: designed the study, analyzed the data, and revised the manuscript. All authors contributed to the article and approved the submitted version.

## Funding

This work was supported by the Department of excellence 2018–2022 initiative of the Italian Ministry of education (MIUR) awarded to the Department of Neuroscience - University of Padua.

## Conflict of Interest

The authors declare that the research was conducted in the absence of any commercial or financial relationships that could be construed as a potential conflict of interest.

## Publisher's Note

All claims expressed in this article are solely those of the authors and do not necessarily represent those of their affiliated organizations, or those of the publisher, the editors and the reviewers. Any product that may be evaluated in this article, or claim that may be made by its manufacturer, is not guaranteed or endorsed by the publisher.

## Supplementary Material

Supplementary Figure 1 Correlation matrices within the caregiver' group (left) and the non-caregivers' group (controls; right) between the components of each standardized questionnaire administered. White spaces indicate that there is no correlation.

Supplementary Figure 2 Correlation between the sleep quality, depression and anxiety's scores predicted by the model vs. the scores really obtained at T2 (*p* < 0.001).
