# Supplementary figures and images for: Depression, Anxiety and Sleep Alterations in Caregivers of Persons With Dementia After 1-Year of COVID-19 Pandemic
Source: Front Psychiatry. 2022 Feb 10;13:826371. doi: 10.3389/fpsyt.2022.826371 (PMC8866969; doi:10.3389/fpsyt.2022.826371)

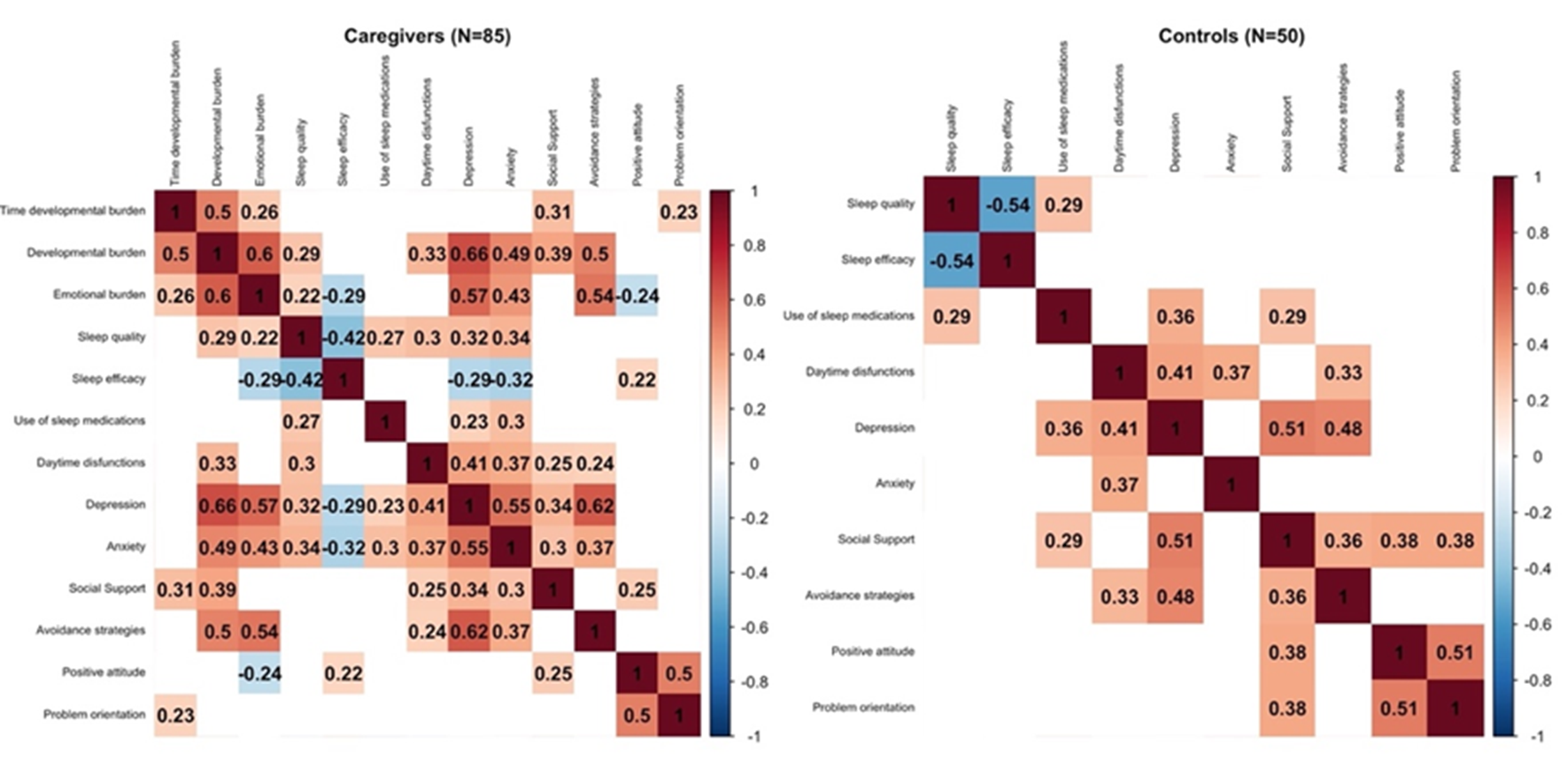

Supplement: Supplementary Figure 1 — Correlation matrices within the caregiver' group (left) and the non-caregivers' group (controls; right) between the components of each standardized questionnaire administered. White spaces indicate that there is no correlation. [file Image_1.PNG]

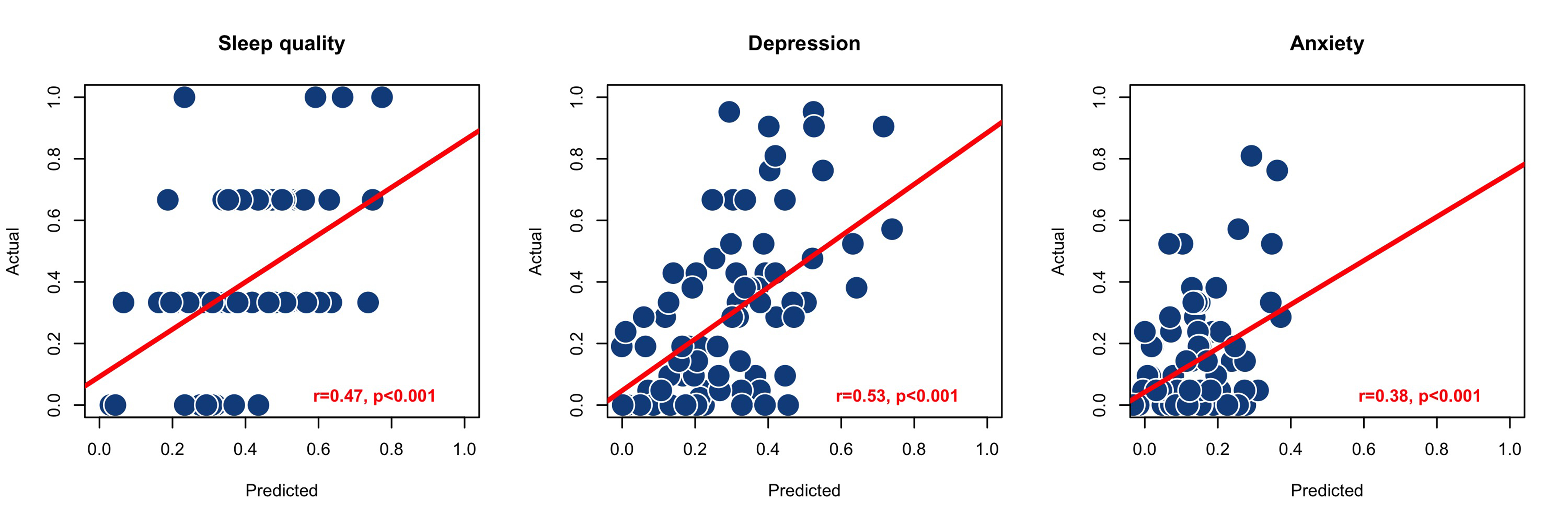

Supplement: Supplementary Figure 2 — Correlation between the sleep quality, depression and anxiety's scores predicted by the model vs. the scores really obtained at T2 (p < 0.001). [file Image_2.JPEG]
